# Painful and painless mutations of *SCN9A* and *SCN11A* voltage-gated sodium channels

**DOI:** 10.1007/s00424-020-02419-9

**Published:** 2020-06-29

**Authors:** Mark D. Baker, Mohammed A. Nassar

**Affiliations:** 1 Blizard Institute, Queen Mary University of London, Whitechapel, London, E1 2AT UK; 2 Biomedical Science, University of Sheffield, Firth Court, Western Bank, Sheffield, S10 2TN UK

**Keywords:** Pain, Dorsal root ganglia, Human mutations, Painful conditions, Voltage-gated sodium channels, Na_v_1.7, Na_v_1.9

## Abstract

Chronic pain is a global problem affecting up to 20% of the world’s population and has a significant economic, social and personal cost to society. Sensory neurons of the dorsal root ganglia (DRG) detect noxious stimuli and transmit this sensory information to regions of the central nervous system (CNS) where activity is perceived as pain. DRG neurons express multiple voltage-gated sodium channels that underlie their excitability. Research over the last 20 years has provided valuable insights into the critical roles that two channels, Na_V_1.7 and Na_V_1.9, play in pain signalling in man. Gain of function mutations in Na_V_1.7 cause painful conditions while loss of function mutations cause complete insensitivity to pain. Only gain of function mutations have been reported for Na_V_1.9. However, while most Na_V_1.9 mutations lead to painful conditions, a few are reported to cause insensitivity to pain. The critical roles these channels play in pain along with their low expression in the CNS and heart muscle suggest they are valid targets for novel analgesic drugs.

## Introduction

Pain is an important warning system to guard against tissue damage and disease. Pathological pain, however, has no warning value and has huge economic, social and personal costs to society. Chronic pain is a global problem affecting up to 20% of the world’s population [9, 50]. Sensory neurons of the dorsal root ganglia (DRG) detect painful stimuli and transmit sensory information to regions of the central nervous system (CNS) that perceive pain. DRG neurons are a heterogeneous population of neurons with distinct functional and histochemical properties [53, 77]. The DRG contains neurons responding to a variety of non-noxious stimuli (such as proprioceptors and low-threshold mechanoreceptors) as well as those responding to noxious stimuli (nociceptors).

Inflammation and nerve injury sensitise DRG neurons and result in decreased pain thresholds and/or intense pain. This can be in part due to increased voltage-gated sodium channel (VGSC) activity resulting in increased excitability of DRG neurons [8, 104]. VGSCs consist of pore-forming α-subunits and auxiliary β-subunits. There are ten cloned α-subunits and 4 β-subunits. The β-subunits modulate the localisation, expression and functional properties of α-subunits [12]. Each α-subunit is composed of four homologous membrane-spanning domains (DI-DIV). Each domain consists of six transmembrane segments (S1-S6) [12]. Different α-subunits have distinct electrophysiological and pharmacological properties [12, 104], and DRG neurons express multiple α-subunits that are essential to their ability to fire action potentials [104].

This review aims to clarify the roles of two VGSC channels expressed selectively, though not exclusively, in primary sensory neurons in pain pathways, and in the light of evidence from genetic mouse models and mutations in man. We discuss the usefulness of these channels as potential drug targets, and suggest that while our present understanding of function has grown more complex, targeting these channels either alone or in combination may still provide a strategy for analgesic development, potentially even for chronic use.

## VGSC as targets for analgesic drugs

There are two reasons why VGSCs are attractive targets for analgesic drugs. Firstly, VGSCs are required for the firing of action potentials in DRG neurons; therefore, blocking their activity will reduce pain signalling in painful conditions even if they were not the primary or only contributor to increased firing. For example, sensitisation of primary transducing channels, like the transient receptor potential (TRP) channels, is often involved in many forms of pathological pain [11, 90, 105]. This sensitisation will lead to greater generator potentials in sensory nerve terminals. However, since VGSCs are required to initiate an action potential in nerve terminals and to allow conduction into the CNS, an effective VGSC blocker can still cancel out the effect of the sensitised TRP channels in nerve terminals.

Secondly, a few of the VGSC α-subunits expressed in DRG are either exclusive to or enriched in DRG neurons that signal pain, with little expression in other DRG neurons, the CNS, skeletal and heart muscles. Blockers for these subunits would therefore be expected to produce analgesia without detrimental side effects. DRG neurons express many of the cloned α-subunits [104]; however, three subunits (Na_v_1.7, Na_v_1.8 and Na_v_1.9) meet the above criteria. Not surprisingly, many pharmaceutical companies are developing and testing subunit specific Na_v_1.7, Na_V_1.8 and Na_V_1.9 blockers as analgesics [38, 128].

The three α-subunits differ in their biophysical properties that determine their role in neuronal excitability [104]. The Na_V_1.9 channel activates over a negative range of membrane potentials close to the resting potential and generates a persistent current. Evidence suggests that it is powerfully regulated by G protein pathways, in a unique way. Therefore, when it is activated, it contributes to setting the resting membrane potentials of neurons expressing it [23]. Na_v_1.7 generates a transient Na^+^ current, but has a relatively slower rate of inactivation near the resting potential (slow closed-state inactivation) allowing the channel to generate persistent currents and making it a so-called threshold channel [22, 25]. Na_v_1.8 has a relatively depolarised activation voltage (~ − 20 mV) compared with Na_v_1.7 and Na_v_1.9 [2, 104]; thus, Na_v_1.8 activation comes after and perhaps subsequent to the activation of Na_v_1.9 and Na_v_1.7 channels. Nonetheless, Na_v_1.8’s depolarised inactivation and more rapid recovery from inactivation allow it to contribute to repetitive firing, for example [104]. This review will focus on Na_V_1.9 and Na_v_1.7 subunits since their biophysical properties allow them both to influence pain thresholds through setting the membrane potential and action potential threshold in DRG neurons.

## Role of Na_v_1.7 in pain

Na_v_1.7 was cloned from PC12 cells in 1997 [118]. At that time, Na_V_1.8 and Na_V_1.3 channels were already under the spotlight and their role in pain was actively being investigated. Na_V_1.8 was cloned in 1996 and its strong expression in medium and small sensory neurons (the sizes of most nociceptors) made it the best and most obvious target for analgesic drug development [2]. The Na_V_1.8 knockout mouse was reported 3 years later and although it showed a pain deficit, its phenotype was compromised by a compensatory upregulation of Na_v_1.7 [3] with clear functional consequences [3, 89]. However, knockdown of Na_V_1.8 by antisense oligonucleotides in adult rats inhibited neuropathic pain [75, 129]. The difference could be due to the timing of the deletion (embryonic versus adult) or the animal model used (mouse versus rat). In contrast to Na_v_1.8, the expression pattern of Na_v_1.3 does not suggest it would be a useful drug target. Na_v_1.3 is expressed throughout the nervous system and its expression is highest during embryonic development and decreases postnatally [121]. However, Na_v_1.3 is the only channel that is re-expressed in DRG following nerve injury and diabetes [122]. This made it a potentially viable target for analgesics. However, mice lacking Na_v_1.3 do not show any deficits in pain phenotype [92].

Na_v_1.7 became the focus of the pain field in 2004 with the publication of two papers [91, 125]. The first paper identified a mutation in *SCN9A* (the gene coding for Na_v_1.7) as the cause for a rare inherited pain condition known as primary erythromelalgia (PEM). PEM symptoms start at early age with episodes of pain in the extremities (usually in the feet) that are triggered by exposure to heat or walking [125]. The second paper reported the complete absence of inflammatory pain in a conditional mouse lacking Na_v_1.7 in most nociceptors [91]. The conditional ablation in nociceptors was achieved using a Cre driver mouse line where Cre is expressed by the Na_v_1.8 promotor [113]. The complete loss of all inflammatory pain and mechanical pressure after ablation of Na_v_1.7 in nociceptors [91] excited the pain field and stimulated drug discovery programmes at several pharmaceutical companies [38, 128]. A conditional mouse was generated because global deletion of Na_v_1.7 in mouse proved to be lethal [91]. Global knockout pups were born alive but failed to feed and died within 24 h. Hand feeding and special husbandry arrangements allow Na_v_1.7 global KOs to survive to adulthood [49]. Inducing Na_v_1.7 ablation in adult mice causes pain deficits without detrimental effects [107].

Remarkably, the symptoms of PEM patients complemented the phenotype of the conditional Na_v_1.7 null mice. While pain can be triggered by mechanical pressure on the feet (walking and exercise), conditional null mutants showed a complete loss of pain to mechanical pressure. While PEM patients showed signs of inflammatory pain (heat, redness and occasionally swelling of the feet), conditional null showed a complete loss of inflammatory pain. This helped support the hypothesis that blocking Na_v_1.7 in humans would significantly reduce pain signalling. However, the mouse study raised significant questions. Firstly, is the role of Na_v_1.7 in pain signalling in humans as critical as it is in mice, or in other words, would the loss or block of Na_v_1.7 in humans lead to the all or none loss of pain seen in mice? Furthermore, if this is the case, then would the loss or block of Na_v_1.7 in humans result in lethality (as it did in mice)? These questions were critical for the validity of Na_v_1.7 as a drug target. These questions were answered in 2006 when it was reported that a loss of function mutation in *SCN9A* causes complete insensitivity to pain (CIP) [19]. In CIP patients, perception of non-noxious touch and warmth is not affected, whereas perception of noxious heat, pressure and injury pain is completely lost. CIP patients confirmed that Na_v_1.7 plays as a critical role in pain signalling in humans as it does in mice. Importantly, loss of Na_v_1.7 did not lead to lethality nor any significant disability (CIP patients are anosmic due to the expression of Na_v_1.7 in the olfactory epithelia [135]). A second heritable painful condition was mapped to a gain of function mutation in *SCN9A* in the same year (2006). Paroxysmal extreme pain disorder (PEPD, initially known as familial rectal pain), was found to be caused by a gain of function mutation in the Na_v_1.7 channel [45]. The four papers between 2004 and 2006 provided very strong evidence that Na_v_1.7 is a critical player in pain signalling, catapulting it to the top of the list of analgesic drug targets. Since then, human geneticists have identified scores of mutations causing PEM, PEPD, CIP and small fibre neuropathy (SFN).

## Primary Erythromelalgia

**Fig. 1:**
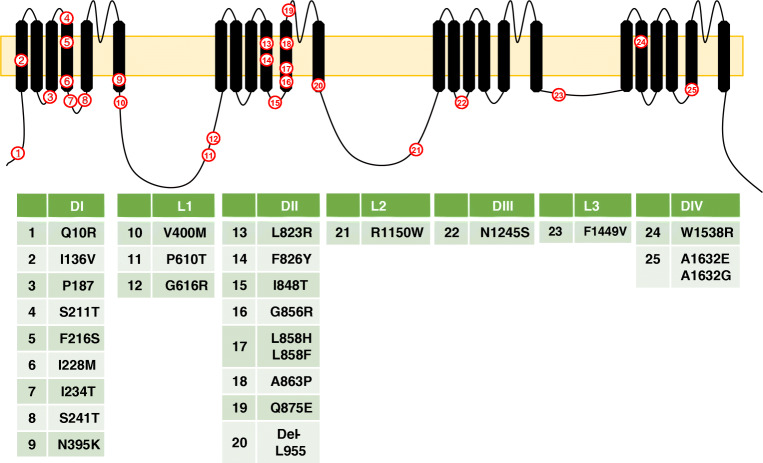
Topological representation of *SCN9A* mutations that cause PEM. A VGSC α-subunit consists of four homologous domains (DI-DIV). Each domain consists of six transmembrane segments. Three intracellular loops (L1-L2) connect the four domains. Note that most mutations are localised to domains I and II. Structures are not drawn to scale

Primary erythromelalgia is an autosomal dominant condition caused by a mutation in the *SCN9A* gene. The condition was first mapped to *SCN9A* in 2004 by Yang et al. [125]. The proband suffered from bilateral episodes of burning pain in their hands and feet that started during their childhood and continued throughout their life. During the attacks, the feet and hands became warm and red. The pain episodes were triggered by exercise or exposure to heat. The proband had the nonsense mutation L858H which is located in the second domain, Fig. 1. Characterisation of the channel’s biophysical properties showed that the mutation shifted the activation voltage about 12 mV in the hyperpolarising direction resulting in a reduced threshold for channel opening and thus increased excitability [24]. Since the first report, several mutations have been reported that cause PEM, listed in Table 1 in chronological order. Symptoms appear early in life although late onset cases have been reported [16, 21]. All PEM mutations cause similar changes to the biophysical properties of Na_v_1.7, involving a shift of the activation voltage to hyperpolarised potentials [30], and where the magnitude of the shift seems to affect the severity of the symptoms [55]. Furthermore, PEM mutations tend to cluster in domains I and II of the channel protein, Fig. 1.

**Table 1:** *SCN9A* mutations that cause PEM in order of their publication

Mutation	Notes on effect	Ref
L858H		[125]
I848T		[33, 125]
L858F		[33]
N395K		[33]
F216S		[33]
P610T		[33]
R1150W		[33]
F1449V		[29]
S241T		[86]
A863P		[59]
I136V	Late onset + reduced intradermal fibre density	[78]
A1632E	Causes PEM and PEPD-like symptoms	[39]
Q10R	Late onset	[55]
V400M	Carbamazepine responsive	[46]
L823R	Shifts fast inactivation to more negative potentials, unusual in PEM	[76]
S211P		[40]
I234T	Sitting and heat trigger pain, one of the largest shift in activation voltage.	[1]
G616R		[16]
Del-L955	Large hyperpolarised shift in activation voltage with a large shift in inactivation voltage in the same direction leading to mild symptoms	[15]
I228M		[41]
Q875E	Severe pain	[110]
Q10K		[72]
V1316A		[42, 123]
A1746G		[21]
W1538R	Described as W1550R in [26]	[21]
A1632T		[35]
L245 V	Incomplete fast inactivation but no shift in activation voltage	[37]
A1632G		[126]
G856R	PEM with impaired limb development	[115]
F826Y		[124]
P187L		[131]
N1245S		[54]

Treatment for PEM patients includes avoidance of the conditions that trigger pain (i.e. heat and physical pressure on the feet). Patients typically resort to foot lifting, cooling feet by fans or immersing them in water or iced water to reduce or relief pain. Although immersion in cold water is effective for mild cases, it can result in ulceration and maceration of foot skin leading to infection [19, 116]. Recently, it has been reported that behavioural therapy reduced dependence on water immersion in PEM patients [67]. There is no consensus on pharmacotherapy. Among effective drugs are non-selective sodium blockers (lignocaine, mexiletine and carbamazepine) [82, 116] which have been shown to inhibit Na_V_1.7 [120, 133].

## Paroxysmal extreme pain disorder

**Fig. 2:**
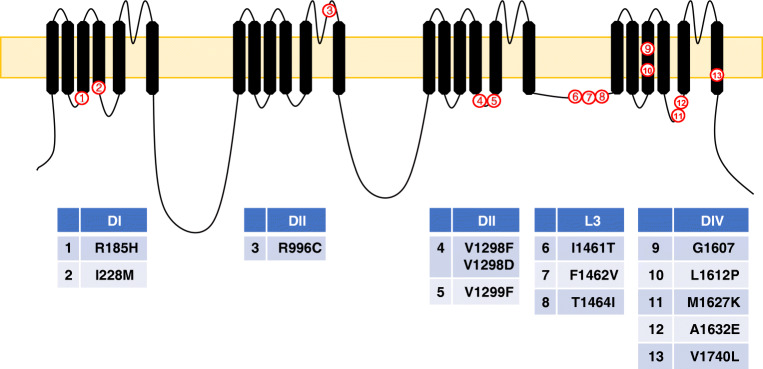
Topological representation of mutations that cause PEPD in the α-subunit of NaV1.7. Note that most mutations are localised in L3 and domains III and IV. Structures are not drawn to scale

Paroxysmal extreme pain disorder (PEPD, formerly known as familial rectal pain syndrome), is caused by gain of function mutations in *SCN9A* that alter the biophysical properties of the Na_v_1.7 channel [45]. There are several similarities between PEPD and PEM. Both are autosomal dominant conditions with symptoms starting early in childhood (PEPD is observed in infants [17]). PEPD is characterised by episodes of severe burning pain in the rectal, ocular and mandibular areas accompanied by flushing of the skin. Pain in PEPD patients is triggered by otherwise innocuous mechanical stimulation (defecating, chewing and yawning) and warmth of the affected areas. However, functional characterisation of mutant Na_v_1.7 channels showed that they have normal activation voltages (unlike PEM mutations). In contrast, PEPD mutations cause a depolarising shift in inactivation voltages with incomplete channel inactivation, leading to a persistent current and increased excitability [7, 45, 119]. Table 2 lists reported *SCN9A* mutations that are found in PEPD patients. PEPD mutations tend to cluster in domains III and IV of the channel protein, Fig. 2. Despite the severity of the pain, PEPD patients responded well to the anti-epileptic drug carbamazepine [117].

**Table 2:** *SCN9A* mutations that cause PEPD in order of their publication

Mutation	Notes on effect	Ref
R996C		[45]
V1298D		[45]
V1298F		[45]
V1299F		[45]
I1461T		[45]
F1462V		[45]
T1464I		[45]
M1627K		[45]
A1632E	Channel displays properties of PEM and PEPD	[39]
G1607R		[17]
I228M	Produces PEM-like symptoms as well	[41]
R185H		[85]
L1612P		[114]
V1740L	Patients suffer from headache	[66]

## Heritable small fibre neuropathy

Small fibre neuropathy (SFN) is caused by damage to thinly myelinated and unmyelinated nerve fibres. SFN is often characterised by late onset, bilateral burning pain to the hands and feet. SFN is also associated with disturbances to autonomic functions like sweating, dryness in the eyes or mouth and disturbance to bowel and bladder functions [117]. Autonomic symptoms are not reported in both PEM and PEPD. Diagnosis is usually confirmed by a decrease in intra-epidermal fibre density (IDFD) in skin biopsies. Several conditions can cause SFN, and these include diabetes and autoimmune disease [52, 112]. About 50% of SFN cases are idiopathic, with no obvious aetiology [112]. The dominant pattern of inheritance of SFN in some cases of idiopathic SFN suggested mutations in a single gene [52, 112]. Mutations in the three peripheral VGSCs, Na_v_1.7 [10], Na_v_1.8 [44] and Na_v_1.9 [63] channels, have been found in heritable SFN cases.

**Fig. 3:**
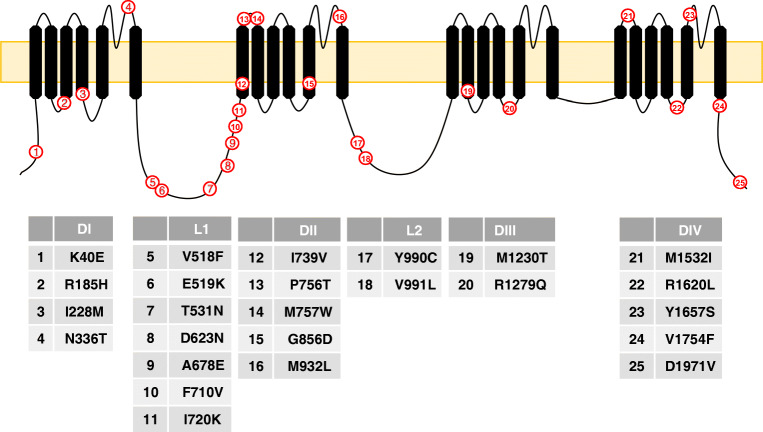
Topological representation of *SCN9A* mutations that cause heritable SFN. Most mutations associated with SFN are found clustered around L1. Structures are not drawn to scale

Characterisation of Na_v_1.7 channels in SFN patients, listed in Table 3, showed that they would cause hyperexcitability [43, 62]; however, it is not clear how this leads to a small fibre neuropathy and why it is of late onset. Na_v_1.7 channel mutations linked to SFN are not localised to a particular region within the channel but many are clustered in the first intracellular loop between domains I and II, Fig. 3. Recently a clinical trial has found lacosamide to be efficacious in reducing pain and well-being of SFN patients with *SCN9A* mutations [27]; however, the effect was linked to subset of *SCN9A* mutations [74].

**Table 3:** Mutations that cause *SCN9A*-linked SFN in order of their publication

Mutation	Notes on effect	Ref
I720K D623N M932L V991L R185H I228M M1532I I739V	Mutations have various impacts on channel properties but all lead to hyperexcitability.	[43]
G856D	Shifts activation voltage to more negative potentials. Shifts fast inactivation to more positive potentials. Causes hyperexcitability.	[62]
K40E N336T V518F E519K T531N A678E F710V W719C I720K P756T M757W Y990C M1230T R1279Q R1620L Y1657S V1754F D1971V	Functional properties uncharacterised	[36]

## Role of Na_v_1.7 in epilepsies

**Fig. 4:**
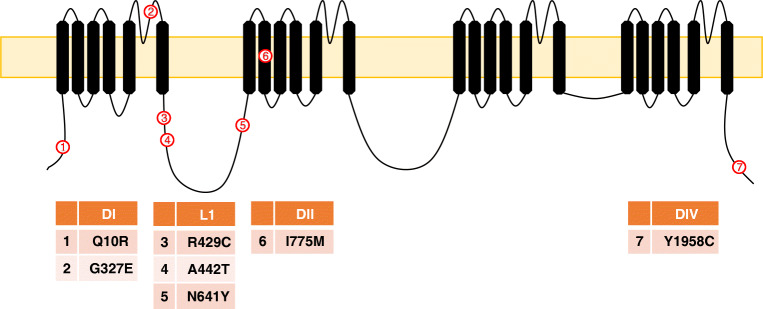
Topological representation of *SCN9A* mutations that are linked to epilepsies

Although the expression of Na_v_1.7 in the human brain is poorly characterised, there is emerging evidence that Na_v_1.7 plays some role in modulating excitability in the brain. It is known that CIP patients suffer from the loss of the sense of smell due to expression of Na_v_1.7 in the olfactory epithelia [135] and a patient with the PEM mutation N1245S displayed high olfactory sensitivity [54]. However, CIP patients are not reported to suffer from brain-related symptoms. Nonetheless, several papers have recently reported mutations in *SCN9A* in patients with various types of epilepsies, Table 4. These mutations are mostly localised to the DI-DII part of the channel, Fig. 4. The above suggests that while a loss of Na_v_1.7 function has no detrimental effect on the brain, altered or increased Na_v_1.7 function does. Therefore, further research is needed to provide insights onto the type of cells that express Na_v_1.7 (types of neurons? any in glia?) in the brain. Furthermore, knock-in models will help to explore how the mutations cause epilepsy rather than act as modifiers to changes in other genes (e.g. *SCN1A*, *SCN2A* and *SCN3A*). Finally, it is intriguing that a few mutations (e.g. Q10R) cause PEM in some patients, and epilepsy in others. This may suggest that variations in the functional expression of other genes or epigenetic changes influence the biological consequences of mutations in Na_v_1.7.

**Table 4:** Mutations that cause epilepsies in order of their publication

Mutation	Notes on effect	Ref
N641Y	Knock-in mice exhibit significantly reduced thresholds to electrically induced seizures.	[109]
Q10R	From patient with febrile seizures plus	[13]
G327E		[127]
G327E	From twin patients with idiopathic focal epilepsy with Rolandic spikes	[81]
I775M	From patient with febrile seizures	[32]
R429C	From patient with febrile seizures plus	[32]
A442T	From patient with genetic epilepsy with febrile seizures plus	[32]
Y1958C	From patient with genetic epilepsy with febrile seizures plus	[132]

## Complete insensitivity to pain

**Fig. 5:**
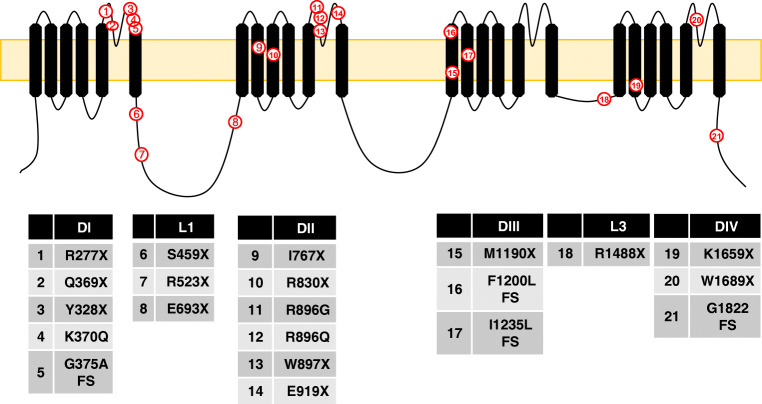
Topological representation of *SCN9A* mutations that cause CIP. Mutations associated with CIP are widely distributed throughout the α-subunit

Complete insensitivity to pain is characterised by loss of all pain sensations throughout patient’s life. *SCN9A* loss of function mutations cause an autosomal recessive CIP [19, 34]. Several mutations have been identified, and most are non-sense mutations causing truncated proteins, Table 5. Most of the mutations are located within domains I and II, Fig. 5. There is recent evidence that the CIP phenotype involves changes to endogenous opioids [87, 97]; however, this was not observed in a rat null model [14].

**Table 5:** *SCN9A* mutations that cause complete insensitivity to pain (CIP) in order of their publication

Mutation	Ref
S459X	[19]
I767X	[19]
W897X	[19]
R277X	[51]
Y328X	[51]
E693X Splice junction mutation intron 23-24	[51]
R830X	[51]
F1200L fs	[51]
R1488X	[51]
K1659X I1235L fs	[51]
W1689X	[51]
R523X	[73]
R896Q	[20]
K370Q G375A fs	[108]
E919X	[96]
M1190X	[106]
G1822 fs	[100]
R896G Q369X	[83]

## Role of Na_v_1.9 in pain

Na_v_1.9 (gene name *SCN11A*) is a tetrodotoxin-resistant (TTX-r), so-called persistent Na^+^ current, with clear evidence for functional expression in nociceptive primary sensory neurons in the dorsal root ganglia (DRG) and trigeminal (e.g. [6, 23]), and the *AH* cells of the myenteric plexus in the gut [18, 102]. The human clone (first named as *SCN12A*, 73% identical with rat *SCN11A*) [68] had initial reported expression in the placenta, spleen, small intestine, spinal cord and brain (potentially neurons and glia). In primary sensory neurons, it has been associated with nerve endings in the tooth pulp and cornea using immunohistochemical methods, and evidence suggests it is found distributed along IB4^+^ axons in the sciatic nerve (e.g. [28, 47, 95]); furthermore, the channel have been located to gut afferents [60, 61] and also to the bladder [101] using electrophysiological methods in gene knockout mice.

**Fig. 6:**
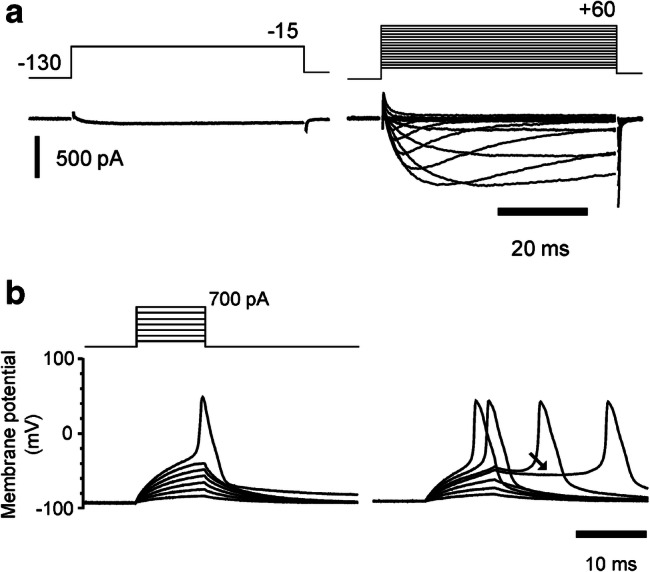
**a** Upregulation of Na_v_1.9 in an Na_v_1.8 knockout neuron, following the introduction of 500 μM GTP-γ-S into the cell interior for 12 min. **b** Upregulation of Na_v_1.9 using intracellular GTP-γ-S gives rise to changes in the firing properties of Na_v_1.8 knockout neuron, with reductions in current and voltage threshold, recorded from a holding potential of − 90 mV. The upregulated current gives rise to plateau potentials (arrow). Reproduced from [6], with permission

The functional properties of the channel currents were first identified in Na_v_1.8 knockout mouse sensory neurons, because under these circumstances, the channel generates the only tetrodotoxin-resistant (TTX-r) Na^+^ current [23, 84]. The channel produces a Na^+^ current in sensory neuron cell bodies that has ultra-slow activation and inactivation kinetics. It gives rise to a persistent, non-inactivating current operating over the negative portion of its activation membrane potential range, allowing it to act as a ‘threshold channel’, and to contribute to setting the membrane potential. Its unusual kinetic properties and negative activation range produce ‘plateau potentials’ that amplify applied or transduced sub-threshold depolarisations and massively prolong them in duration (Fig. 6). It is worth noting that Na_v_1.9 has activation kinetics that are too slow to directly contribute to impulse firing.

Intracellular dialysis of the non-hydrolysable GTP analogue, GTP-γ-S, upregulated the Na_v_1.9 current with no changes in current kinetics, recorded in voltage-clamp. It was also found that at a membrane potential of near − 60 mV, functional upregulation of the current can cause sensory neurons to fire rhythmically and spontaneously, at low frequency (Fig. 6) [6]. The current could be upregulated following the activation of ATP receptors, deduced to be P2Y, operating through a probable Gq/11 pathway and PKC [5, 6], and such a pathway has later been confirmed to be a contributor to modifying the firing properties of gut afferents.

There are several mouse knockouts of *SCN11A* reported in the literature and these have been associated with an elimination of the GTP-γ-S-upregulated current in primary sensory neurons [94] and a complementary reduction in forms of inflammatory pain following exposure to PGE2 [4] and including the formalin and CFA tests [99]—consistent with a role of Na_v_1.9 in inflammatory pain in both the skin and gut. A likely role for Na_v_1.9 in the control of normal gut motility, attributable to altered plexus function, seems consistent with the effects of mutation in human carriers and gain of function is associated with constipation (e.g. [69]). With these facts in mind, it may be possible to understand the defects in pain signalling found in humans with rare, heritable mutations in *SCN11A*.

## Painful and painless Na_v_1.9 channelopathies

About 20 mutations have been reported for *SCN11A*, Table 6. All follow a dominant inheritance pattern. Most mutations have been confirmed to lead to a gain of function. No loss of function mutations have been reported to date which could be because such mutations cause mild or no effect on pain signalling in humans (given the phenotype of knockout mice, it is very unlikely that human loss of function mutation causes lethality). It is also possible that the loss or reduction of inflammatory pain may mean such individuals are unlikely to have a reason to visit the doctor!

**Table 6:** *SCN11A* mutations that cause painful and painless human conditions. Mutations causing painless condition are associated with big shifts in either their activation or inactivation voltages

Mutation	Notes on effect	Ref
L811P	CIP Activation voltage shifted by − 26 mV	[79]
R225C A808G	Episodic pain Increase current density No effect on activation and inactivation voltages	[130]
I381T K419N A582T A681D A842P L1158P F1689L	Painful neuropathy I381T: activation voltage shifted by − 6.7 mV L1158P: activation voltage shifted by − 6.9 mV	[63]
G699R	Painful neuropathy Activation voltage shifted by − 10.1 mV	[57]
V1184A	Episodic pain Activation voltage shifted by − 17 mV	[80]
R222S R222H	Episodic pain	[93]
R222H	Episodic pain Activation voltage shifted by − 6.4 mV	[58]
L1302F	CIP	[98]
N1169S I1293V	PEM-like pain	[131]
L396P	CIP Deactivation voltage shifted by − 22.8 mV	[70]
L1302F	CIP	[64]
N820Y	Painful neuropathy	[48]
N816K	Episodic pain	[65]

The persistent nature of Na_v_1.9 currents and the negative activation voltage dependence make the channel functionally unique. It is proposed to act as a threshold channel in peripheral nociceptors, so gain of function mutations associated with facilitated activation would be expected to give rise to painful neuropathy, because the threshold for action potential generation is reduced. Indeed, *SCN11A* mutations result in two painful conditions, familial episodic pain [65, 80, 93, 130] and painful small fibre neuropathy [48, 57, 63]. In familial episodic pain, painful episodes centre on regions on the arms and legs; in addition, there are age-related decreases of pain, suggesting real age-related changes in gene expression. Painful episodes (lasting 10 s of minutes) are associated with rainy days, cold temperature and commonly also fatigue; some are associated with gut motility symptoms. Further, drugs acting as NSAIDs or anti-pyretics, such as ibuprofen, appear to be able to ameliorate these symptoms. Patients with *SCN11A*-related small fibre neuropathy experience pain, tingling and numbness in their arms and legs. Patients may experience diarrhoea which is consistent with expression of Na_v_1.9 in the gut [130].

**Fig. 7:**
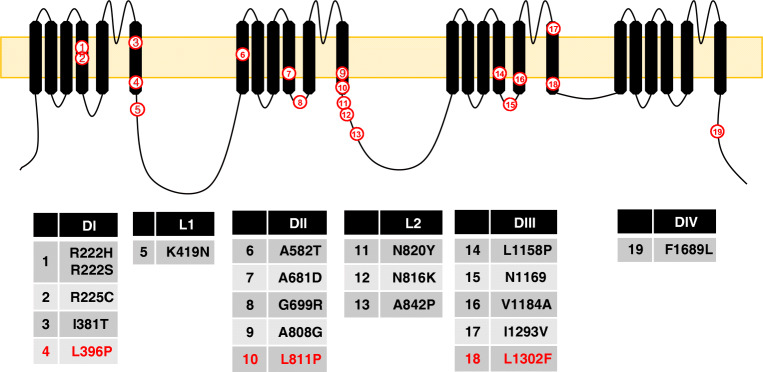
Topological representation of *SCN11A* mutations. Notice that all CIP causing mutations (red) are located in the transmembrane segment 6

Surprisingly, a few *SCN11A* gain of function mutations cause a complete insensitivity to pain [70, 79, 98]. Several possible explanations for how enhanced channel function leads to reduced neuronal excitability have been suggested [31], although arguments concerning modifications of channel-gating kinetics as the primary cause seem incomplete and are based on voltage-clamp recordings whose interpretation may not be straightforward. It is thought that increased activation/inactivation-gating overlap (or window current) depolarise the Na_v_1.9 expressing neurons. This prolonged depolarisation causes rapidly gating Na^+^ channels (e.g. Na_v_1.7 and Na_v_1.8) to enter the inactivated state [64]. Since these channels underpin action potential generation and propagation, the depolarizing block of Na_v_1.7 and Na_v_1.8 in nerve endings leads to an overall decrease in excitability. It was noted that the mutations that lead to CIP are those that produced the largest shift in the activation threshold of the channel, whereas those that lead to familial episodic pain and painful small fibre neuropathy cause smaller shifts, Table 6 [31]. Also of note, *SCN11A* CIP mutations are all localised to transmembrane segment 6, Fig. 7. However, several issues are difficult to reconcile with the above explanation for the painless phenotype. Firstly, Na_v_1.9 is expressed in the IB4^+^ subset of neurons and not in all DRG neurons (at least in rodents). Therefore, a depolarising block in this subset of neurons alone is not expected to cause a complete loss of pain. Second, Na_V_1.8 which is expressed in most nociceptors (i.e. in same neurons as Na_v_1.9) is a channel known to operate at more depolarised membrane potentials and can maintain excitability, even in the face of a depolarised membrane potential [56, 103, 111, 134].

## Concluding remarks

In the past 20 years, mouse models and human genetics have confirmed that Na_v_1.7 and Na_v_1.9 play critical roles in pain signalling. The link between genotype and phenotype for mutations in both channels is poorly understood. Symptoms manifest in the extremities (mainly in the feet/legs) in most human conditions. In Na_v_1.7 channel mutations, there is a link between mutations that cause enhancement of activation to PEM and mutations that cause incomplete inactivation to PEPD. The physiological or microanatomical basis for these associations in terms of nerve ending function is only partly understood. In Na_V_1.9 channel mutations, a clear understanding of why most gain of function mutations cause painful conditions while those affecting segment 6 cause insensitivity to pain is also lacking, although insensitivity to pain is hypothesised to be caused by reduced excitability due to a depolarising block [121, 122].

Nonetheless, available evidence confirms a critical role for both channels in pain earning them a position in the list of potential drug targets. Ablation of Na_v_1.8 and Na_v_1.9 in mice does not lead to lethality or any observable detrimental effects. Ablation of Na_v_1.7 in human [19] and in adult mice [107] does not lead to lethality. Reassuringly, there are no reported respiratory or behavioural abnormalities as a result of the absence of any one of these three channels in mouse and human. Yet important challenges for VGSC blockers in chronic pain remain, developing subunit-specific blockers being the first. This is important considering that VGSC blockers would need to be administered regularly to treat chronic pain and perhaps at higher doses for breakthrough pain, when pain is severe. Therefore, VGSC blockers would need to be safe for long-term administration. The development of specific and effective small molecule inhibitors of Na_v_1.7 is still elusive, despite efforts by several pharmaceutical companies [38, 71, 128]. The second challenge is the extent of Na_v_1.7 inhibition required for analgesia in vivo. Given that CIP carriers have normal pain phenotype, an Na_v_1.7 blocker may need to reduce channel activity to a level well below 50% to produce analgesia.

The contribution of the endogenous opioid system to the phenotype of the *SCN9A* CIP raises the question of whether the CIP phenotype is a direct consequence of the loss of Na_v_1.7 [87, 97]. Several papers have provided complementary evidence that the loss of Na_v_1.7 reduces the excitability of DRG neurons per se. Deletion of Na_v_1.7 causes an increase in action potential threshold in small-diameter DRG neurons [107]. Deletion of Na_v_1.7 causes a major decrease in DRG neuron responsiveness to the VGSC opener veratridine [89]. Mechanically evoked spiking of C-fibres in the skin-nerve preparation was reduced in Na_v_1.7 KO mice [49]. No changes in the expression of other VGSC channels were reported in the Na_v_1.7 KO mouse to contribute to the observed reduced excitability of DRG [107]. Furthermore, pain deficits in a rat model of Na_V_1.7 deletion were not reversed by the opioid receptor antagonist naloxone [14]. Therefore, although an increase in spinal cord opioid synthesis would reduce signal transmission at the first synapse in CIP patients, the loss of Na_V_1.7 has a profound effect on the excitability of DRG neurons (i.e. expected to affect the initiation of the pain signal in the periphery).

The lack of reported Na_v_1.9 loss of function mutations may indicate that its loss does not lead to a major phenotype in humans, or at least does not make people go to the doctor, raising doubts as to whether a blocker would lead to a major analgesic effect. Finally, considering that nociceptors express at least two of the peripheral VGSC subunits (Na_v_1.7, Na_v_1.8 and Na_v_1.9), an effective analgesic strategy may ultimately result from a combination of blockers against these subunits to have additive and synergistic effects on nociceptors. The effectiveness of various drug combinations to reduce neuronal excitability can only be measured in DRG neurons because they express the target VGSCs at biologically relevant levels. Equally important, for the evaluation of any drug combination, is the potential effects on non-nociceptors as well as nociceptors. We recently described a relevant assay [88] and provided proof-of-concept data that showed a combination of Na_v_1.7 and Na_v_1.8 blockers produced a reduction in the excitability of DRG neurons close to that measured in Na_v_1.7 KO [89]. Changing the constituents and doses in VGSC blocker combinations may enable clinicians to manage chronic pain safely over the long term.
